# Supplementary material for: Cellular senescence in malignant cells promotes tumor progression in mouse and patient Glioblastoma
Source: Nat Commun. 2023 Jan 27;14:441. doi: 10.1038/s41467-023-36124-9 (PMC9883514; doi:10.1038/s41467-023-36124-9)
Supplement: Supplementary file 1 — Supplementary Information [file 41467_2023_36124_MOESM1_ESM.pdf]

Cellular senescence in malignant cells promotes tumor progression in mouse and patient Glioblastoma

Supplementary information

Supplementary Figure 1

a

#Tumor	Type	Age	Sex	Molecular alterations/genes	Molecular alterations/chromosomes	CDKN2A status	%SA-β-gal area
1	GBM	65	F	hTERT C228T	7 gain; 9p loss; 10 loss	deleted	1.5
2	GBM	51	M	EGFR ampl.; hTERT C228T; PTEN mut.; EGFR mut.	7 gain; 9p partial loss; 10 loss; 19 gain	WT/normal	3.17
3	GBM	70	F	no alteration	no alteration	WT/normal	0.50
4	GBM	51	M	hTERT C250T; MDM2 ampl.	no alteration	WT/normal	0.27
5	GBM	71	M	hTERT C228T; TP53 mut.; RB1 mut.; CDK4 ampl.	7 gain; 10 loss	WT/normal	1.95
6	GBM	85	F	hTERT C228T; TP53 mut.; PTEN mut.; PDGFRa ampl.	7 gain; 9p partial loss; 10 loss	deleted	0.06
7	GBM	77	M	hTERT C228T; TP53 mut.; EGFR ampl.; MGMT methyl.	7 gain; 9p loss; 10 loss	deleted	0.35
8	GBM	51	M	hTERT C228T; TP53 mut.; PTEN mut.; ATRX mut.; MDM2 ampl.; MGMT ampl.; CDK4 ampl.	7 gain; 10 loss	WT/normal	0.59
9	GBM	64	F	TP54 mut.; MGMT methyl.	1p partial del.; 7 gain; 9p loss; 10 loss	deleted	1.32
10	GBM	52	F	EGFR ampl.; hTERT C250T; TP53 mut.	7 gain; 9p partial loss; 10 loss; 9p19q gain	deleted	0.16
11	GBM	78	M	hTERT C250T; MGMT methyl.; FUBP1 mut.; ATRX mut.; NF1 mut.	9p loss; 10 loss	heterozygous	3.25
12	GBM	61	F	MGMT methyl.	7 gain; 4q partial loss; 10 loss; 13q partial loss; 19 gain	WT/normal	1.27
13	GBM	43	M	no alteration	7 gain; 9p partial loss; 10 loss; 14q loss; 15q partial loss; 17q partial loss	WT/normal	0.04
14	GBM	63	M	EGFR ampl.; hTERT C228T; MGMT methyl.	1 partial loss; 7 gain; 9p partial loss; 10 loss; 19 gain	deleted	0.05
15	AA IV	67	M	IDH1 mut.; TP53 mut.; ATRX mut.; NF1 mut.; PTEN mut.; PMS2 mut.; CDKN2A H83Y	7 gain; 9p partial loss; 10q partial loss	mutated	1.56
16	AA III	47	M	IDH1 mut.	NS	NS	0.15
17	AA III	44	F	IDH1 mut.; TP53 mut.	1p gain; 7q gain; 19q gain	WT/normal	0.57
18	A II	26	M	IDH2 mut.; TP53 mut.; ATRX mut.	no alteration	WT/normal	0.51
19	AA III	32	F	IDH1 mut.; TP53 mut.; ATRX mut.	1p partial del.	WT/normal	1.63
20	O III	61	M	IDH1 mut.; hTERT C250T; CIC mut.	1p19q codel.; 9p loss	heterozygous	1.97
21	O III	36	M	IDH1 mut.; hTERT C250T; CIC mut.	1p19q codel.; 8 loss	WT/normal	0.36
22	O III	50	F	IDH1 mut.; hTERT C228T; CIC mut.	1p19q codel.	WT/normal	0.78
23	O III	44	M	IDH1 mut.; hTERT C228T; PIK3CA mut.	1p19q codel.; 6p partial loss; 9p partial loss; 15q loss; 18 loss; 22q partial loss	deleted	0.06
24	O III	35	M	IDH1 mut.; hTERT C228T; PTEN mut.	1p19q codel.	WT/normal	6.78
25	O III	31	F	IDH1 mut.; hTERT C228T	1p19q codel.; 14q partial loss	WT/normal	0.04
26	O III	37	M	IDH2 mut.; hTERT C250T; CIC mut.	1p19q codel.	WT/normal	0.26
27	O II	39	F	IDH1 mut.; CIC mut.	1p19q codel.	WT/normal	0.26
28	O II	43	M	IDH2 mut.; hTERT C228T	1p19q codel.	WT/normal	0.26

recurrence

senescence categories
>1%
0.1-1%
≤0.1%

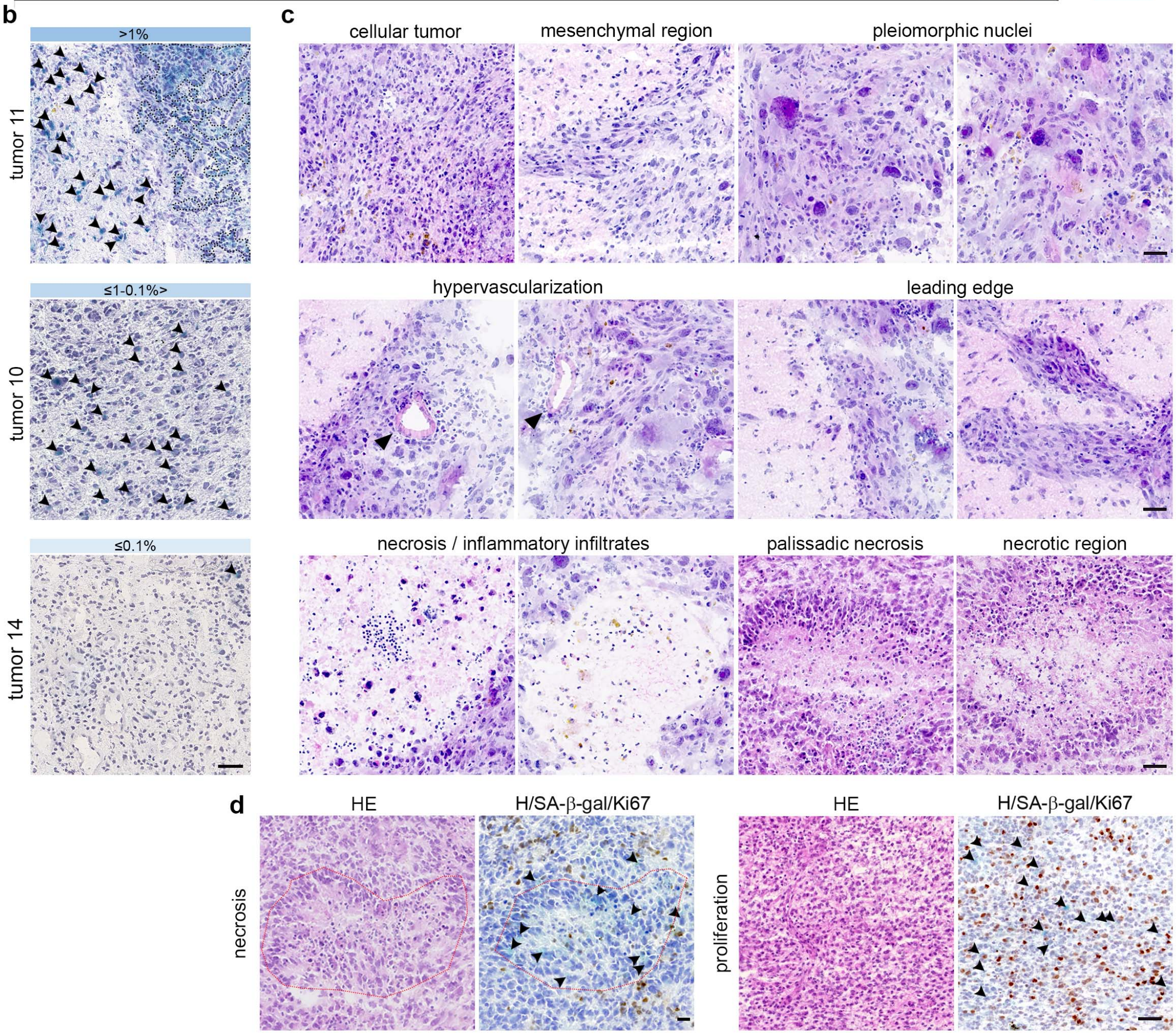

Supplementary Figure 1. Identification of senescent cells in patient and mouse gliomas. (**a**) Table recapitulating the patient resected diffuse gliomas stained for SA-β-gal and annotated for their glioma type, age, sex, molecular alterations and percentage of SA-β-gal area over the tumor area. Recurrent gliomas are highlighted in grey.

(**b**) Representative SA-β-gal staining (blue) of the 3 categories of senescence in patient gliomas. Sections are counterstained with hematoxylin (H) (n=28 independent patient GBMs).

(**c**) Representative Hematoxylin and Eosin (HE) staining on mouse GBM cryosections. The mouse GBM model recapitulates the patient GBM histological features. Note that all mouse or patient GBMs included in this manuscript have been counterstained in HE.

(**d**) Representative HE and SA-β-gal/Ki67 staining on adjacent mouse GBM cryosections highlighting the presence SA-β-gal+ cells in proliferative and necrotic areas (n=8 independent mouse GBMs).

Scale bars, **b**: 50 µm; **c**: 20 µm; **d**: 100 µm. amp: amplification; del.: deletion; methyl.: methylation; mut.: mutation; NS: not specified. Arrowheads point to SA-β-gal+ cells. Dashed lines highlight SA-β-gal+ area in **b,** and necrotic area in **d**. Raw data are provided as a Source Supplementary Data file.

Supplementary Figure 2

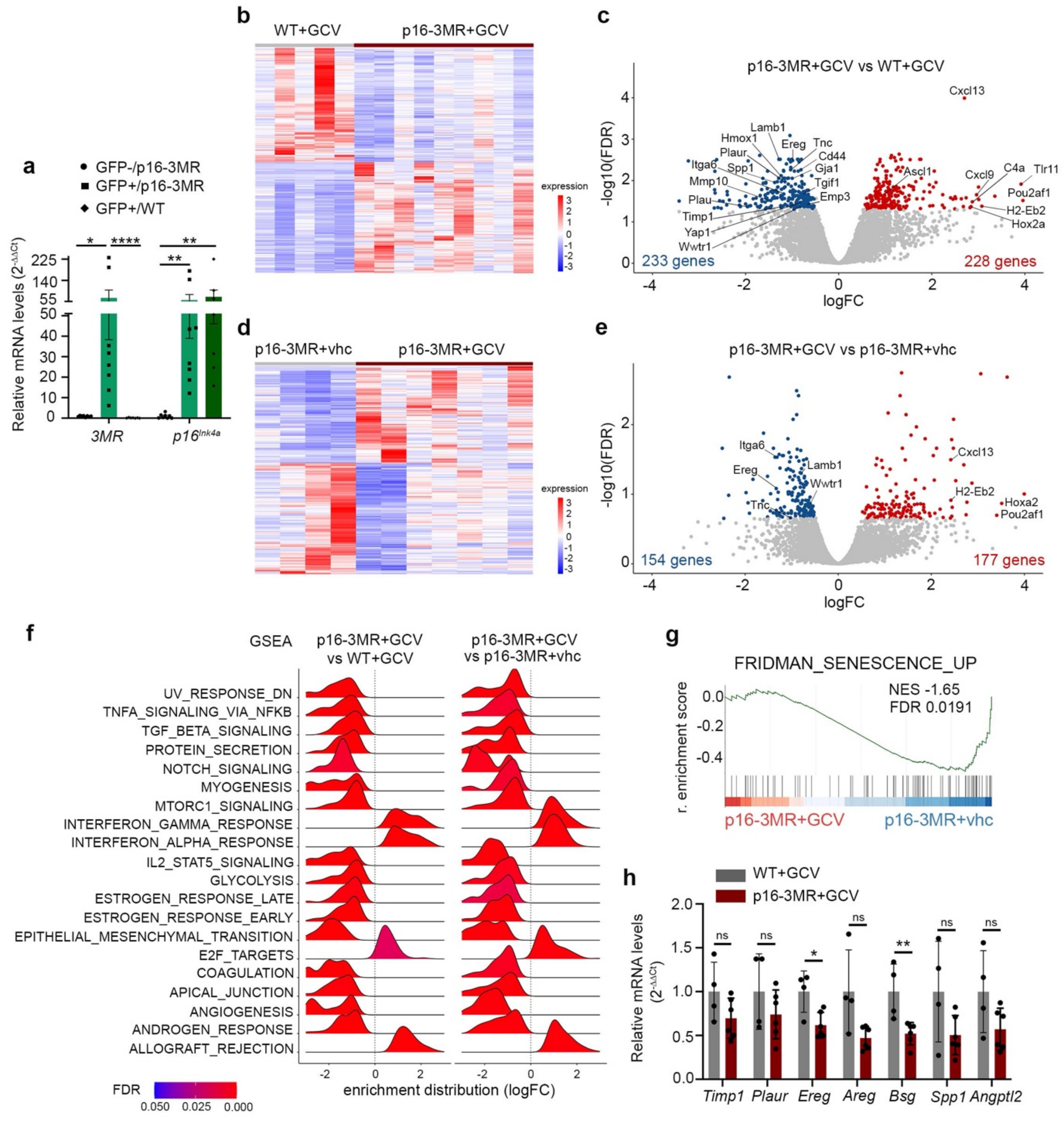

Supplementary Figure 2. Senescent cells partial removal increases the survival of GBM bearing mice

(**a**) Relative transcript levels shown as ratios of normalized values of tumor samples (GFP+, n=7) from WT and p16-3MR transgenic mice (GFP+, n=8) over the surrounding parenchyma from the same transgenic animals (GFP-, n=8). Data are represented as the mean ± SEM. Statistical significance was determined by a Kruskal Wallis test with a Dunn's multiple comparisons correction (* $p<0.05$; ** $p<0.01$; **** $p<0.0001$).

(**b**) Heatmaps representing bulk RNAseq analysis of the differentially expressed (DE) genes (FDR<0.05; logFC>0.5) between p16-3MR+GCV (n=9) compared with WT+GCV (n=5) GBMs collected at the end points of the mice.

(**c**) Volcano plots of the DE genes (FDR<0.05; logFC>0.5) between p16-3MR+GCV (n=9) compared with WT+GCV (n=5) GBMs collected at the end points of the mice.

(**d**) Heatmaps representing bulk RNAseq analysis of the DE genes (FDR<0.05; logFC>0.5) between of p16-3MR+GCV (n=7) compared with p16-3MR+vhc GBMs (n=4) collected at the end points of the mice.

(**e**) Volcano plots of the DE genes between p16-3MR+GCV (n=7) compared with p16-3MR+vhc GBMs (n=4) collected at the end points of the mice. Annotated genes are common to those in B.

(**f**) GSEA ridge plots of the significant representative Hallmark gene lists common to the two RNAseq analysis (**a**, **b** and **c**, **d**).

(**g**) GSEA graph representing the enrichment score of the Fridman senescence pathway in p16-3MR+GCV compared with p16-3MR+vhc GBMs.

GCV: ganciclovir; FDR: false discovery rate; NES: normalized enrichment score; r. enrichment score: running enrichment score.

(**h**) Relative transcript levels shown as ratios of normalized values of p16-3MR+GCV GBMs (n=6) over WT+GCV GBMs (n=4). Data are represented as the mean ± SD. Statistical significance was determined by Wilcoxon-Mann-Whitney test (**, $p<0.01$, ns, not significant). Raw data are provided as a Source Supplementary Data file.

Supplementary Figure 3

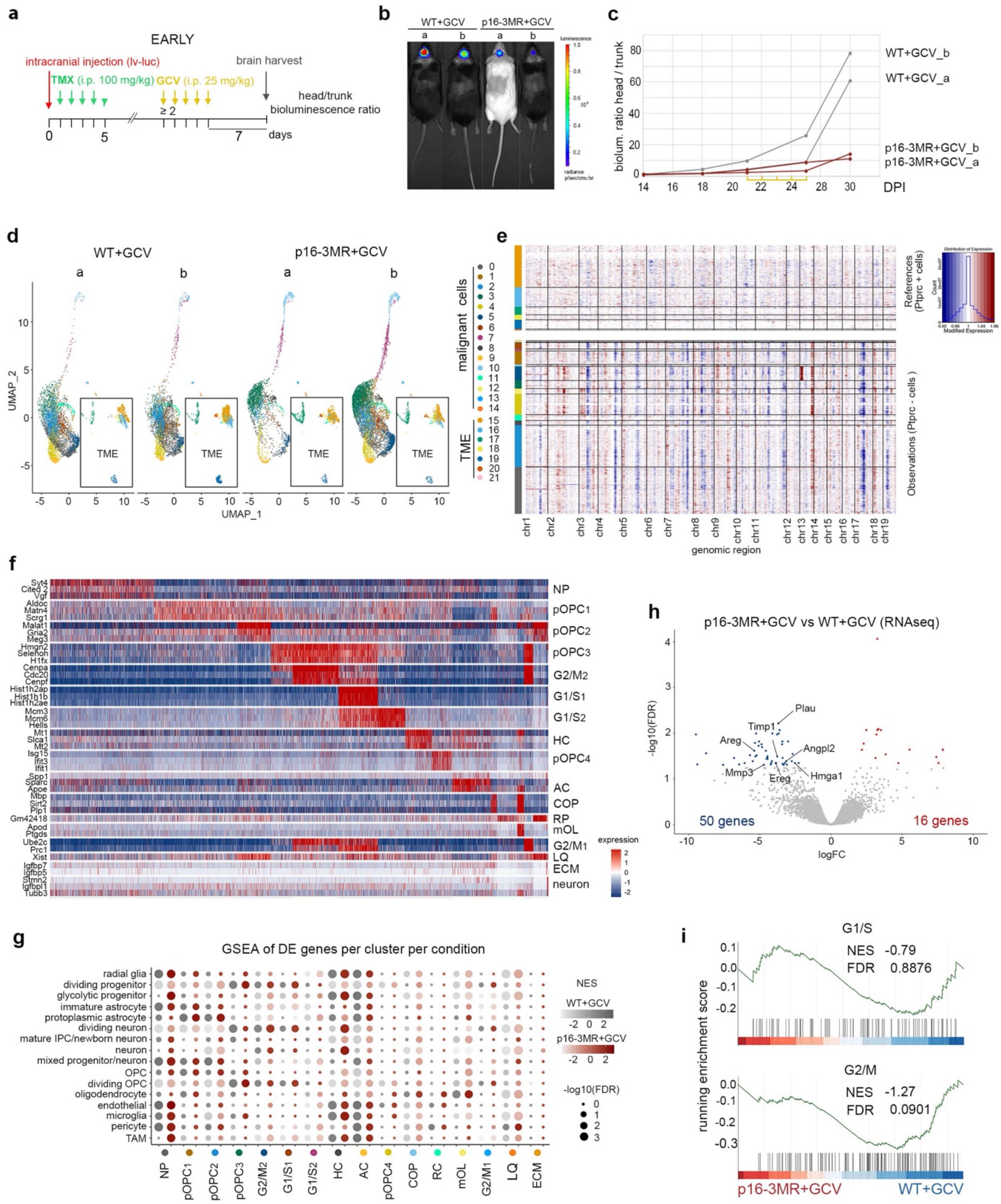

Supplementary Figure 3. Identification of p16$^{Ink4a\ Hi}$ cells in a subset of malignant cells

(**a**) Timeline of the mouse GBMs generation for scRNAseq at the early timepoint.

(**b**) *In vivo* bioluminescence imaging of WT+GCV and p16-3MR+GCV GBM-bearing mice.

(**c**) Graph representing the head to body ratio bioluminescence of GBM-bearing mice over time. Yellow lines correspond to GCV injections.

(**d**) UMAP plots of WT+GCV and p16-3MR+GCV GBM cells per biological sample at a 0.5 resolution and annotated malignant cells and cells from the tumor microenvironment (TME).

(**e**) Heatmap representing inference of chromosomal copy number variations (CNVs) in WT+GCV GBMs with cells as rows, grouped in clusters, and genes as columns, ordered according to chromosome.

(**f**) Heatmap of the top 3 differentially expressed (DE) genes (FDR<0.05; avlogFC>0.25) in malignant clusters (0.6 resolution) in WT+GCV GBMs. When the same gene is DE in more than one cluster, it appears only once.

(**g**) GSEA dot plots of DE genes (FDR<0.05; avlogFC>0.25) in WT+GCV (grey dots) and p16-3MR+GCV (red dots) GBMs of gene lists from Bhaduri *et al.*[1] (Supplementary Table 1).

(**h**) Volcano plots of the DE genes between of p16-3MR+GCV (n=4) compared with WT+GCV GBMs (n=4).

(**i**) GSEA graph representing the enrichment score of the cycling pathways (Weng *et al.*[2], 2019; Supplementary Table 1) in p16-3MR+GCV (n=4) compared with WT+GCV (n=4).

d-g: analysis performed from scRNAseq data as shown in **a-c**. **h** and **i**: analysis performed from bulk RNAseq data at the early time point of the mice. GCV: ganciclovir; TMX: tamoxifen; i.p.: intraperitoneal; lv-luc: lentivirus-luciferase; UMAP: uniform manifold approximation and projection; chr: chromosome; FDR: false discovery rate; NES: normalized enrichment score. Raw data are provided as a Source Supplementary Data file.

Supplementary Figure 4

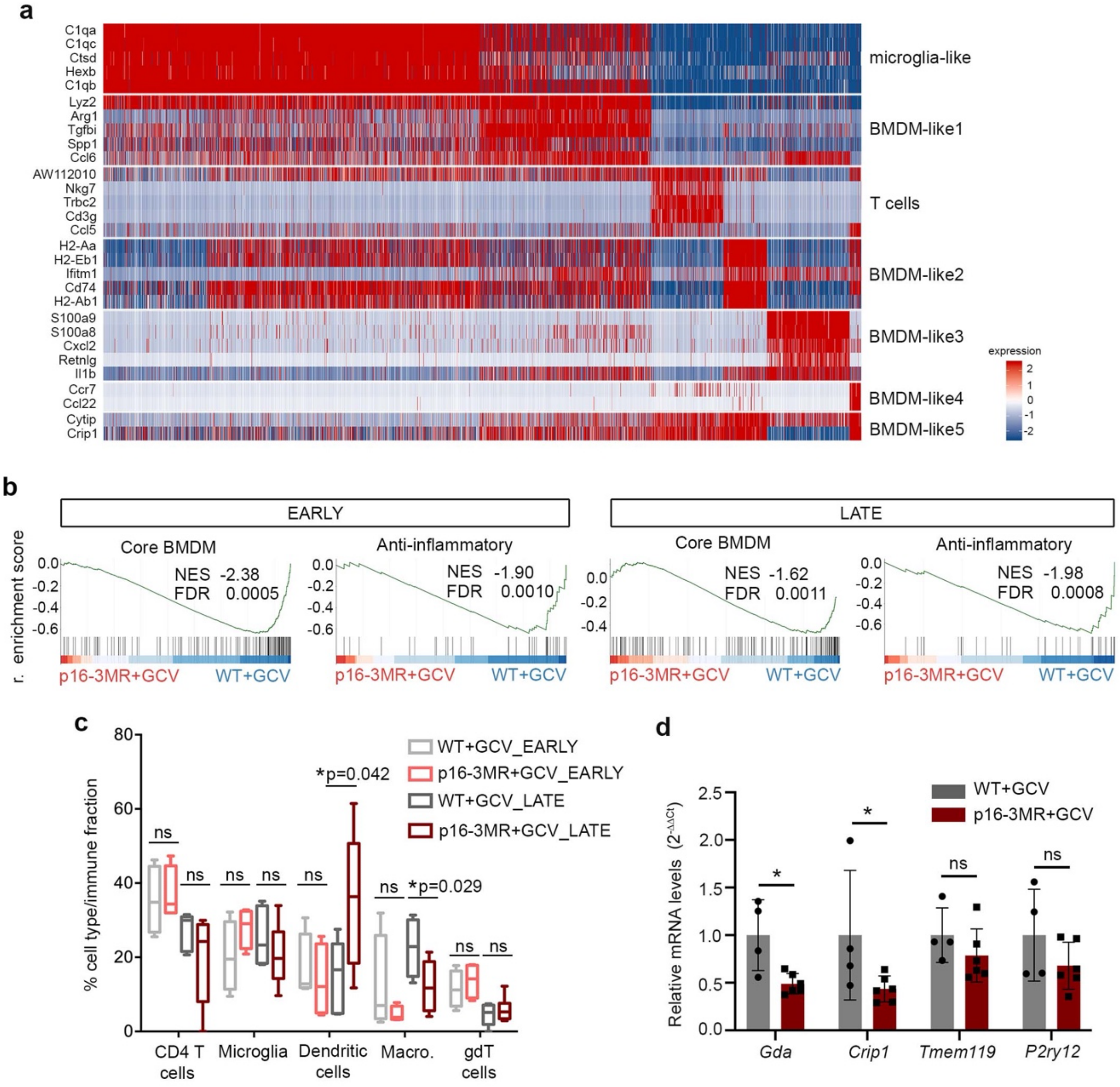

Supplementary Figure 4. Modulation of the immune compartment following p16$^{Ink4a\ Hi}$ cells partial removal

(**a**) Heatmap of the top 5 differentially expressed (DE) genes (FDR<0.05; avlogFC>0.25) in *CD45*+ clusters (0.5 resolution) in WT+GCV GBMs. When the same gene is DE in more than one cluster, it appears only once.

(**b**) GSEA graphs representing the enrichment score of core bone marrow-derived macrophages (BMDM) and Anti-inflammatory pathways (Bowman *et al.*[3]; Darmanis *et al.*[4]; Supplementary Table 1) in p16-3MR+GCV compared with WT+GCV GBMs at the early and late timepoints. Analysis performed from bulk RNAseq data.

(**c**) Bar plot representing the estimation of the abundance of immune cell types in WT+GCV (n=4 for the early time point and n=5 for the late time point) and p16-3MR+GCV (n=4 for the early time point and n=9 for the late time point) GBMs using CIBERSORT (reference data set GSE124829) at the early and late time points. Analysis performed from bulk RNAseq data. Statistical significance was determined by Wilcoxon-Mann-Whitney test (ns, not significant; *, p<0.05).

(**d**) Relative transcript levels shown as ratios of normalized values of p16-3MR+GCV GBMs (n=6) over WT+GCV GBMs (n=4) collected at the late timepoint. Data are represented as the mean ± SD and statistical significance was determined by Wilcoxon-Mann-Whitney test (*, p<0.05; ns, not significant).

FDR: false discovery rate; NES: normalized enrichment score; macro.: macrophages; GCV: ganciclovir. Raw data are provided as a Source Supplementary Data file.

Supplementary Figure 5

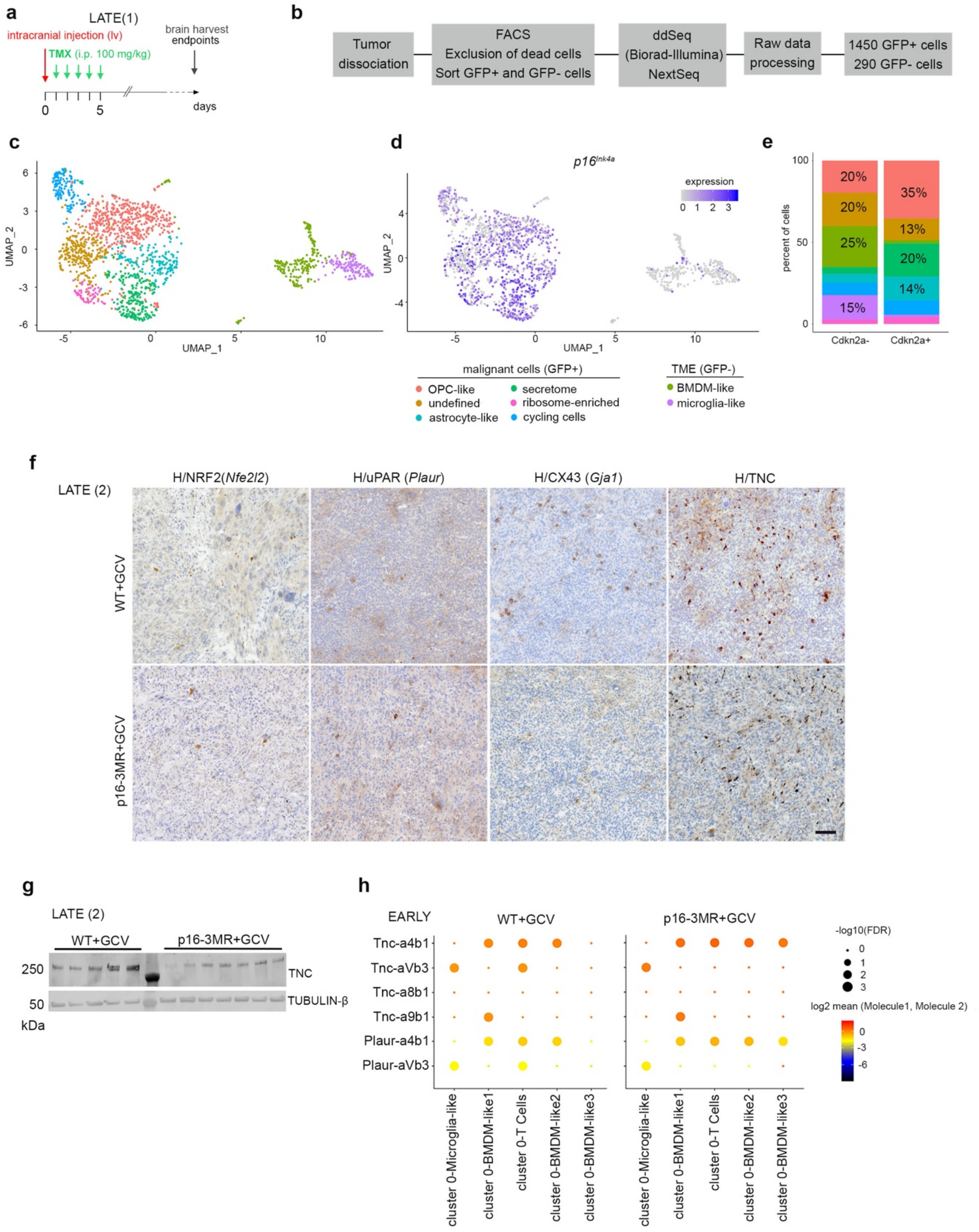

Supplementary Figure 5. Identification of NRF2 activity and its putative targets in p16^{Ink4a} ^{Hi} malignant cells

(**a**) Timeline of the mouse GBM generation for scRNAseq at the late timepoint (LATE(1)).

(**b**) Scheme of the scRNAseq experiment.

(**c**) UMAP plots of GBM cells and annotated cell type at a 0.6 resolution.

(**d**) UMAP plots of the expression of *p16^{Ink4a}* in GBM cells.

(**e**) Barplot representing the percentage of cells per cluster positive and negative for *p16^{Ink4a}* expression.

(**f**) Low magnification of representative immunohistochemistry (IHC, brown) counterstained with hematoxylin (H, purple) on mouse GBM cryosections at the late timepoint (NRF2: WT+GCV, n=7; p16-3MR+GCV n=7; uPAR: WT+GCV, n=3; p16-3MR+GCV, n=3; CX43: WT+GCV, n=5; p16-3MR+GCV, n=6; TNC: WT+GCV, n=5; p16-3MR+GCV, n=7 independent mouse GBMs).

(**g**) Western blot (WB) for TNC from independent WT+GCV (n=5) and p16-3MR+GCV (n=7) GBMs collected at the late timepoint. Each lane corresponds to one GBM. TUBULIN-β was taken as a loading reference. Quantification of the WB is shown in Fig. 5l.

(**h**) Dot plot representing ligand-receptor interactions between the cluster 0 and the immune clusters in the scRNAseq data at the early timepoint using CellPhoneDB. The colors indicate the mean expression of the ligand-receptor complexes.

Scale bar, **f**: 40 µm. GCV: ganciclovir; TMX: tamoxifen; FACS: fluorescence-activated cell sorting; UMAP: uniform manifold approximation and projection; TME: tumor microenvironment. Raw data are provided as a Source Supplementary Data file.

Supplementary Figure 6

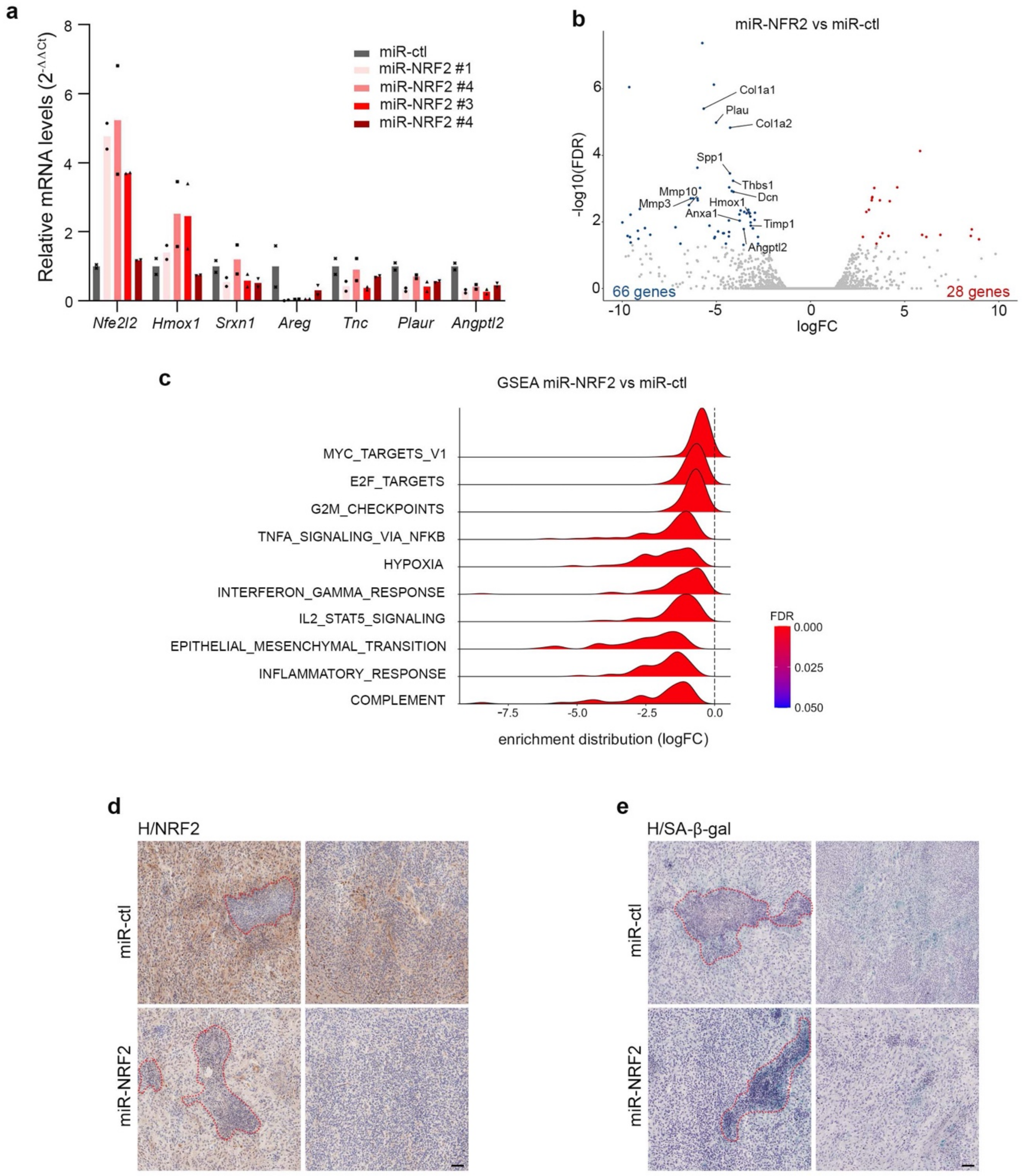

Supplementary Figure 6. Knockdown of NRF2 in malignant cells recapitulates most features of the senolytic treatment

(**a**) Relative transcript levels shown as ratios of normalized values of GL261 cells expressing miR-NRF2 over miR-ctl. The graph shows two independent experiments performed in duplicate. miR-NRF2 #4 reduces the expression of all the NRF2 targets (canonical targets: *Hmox1* and *Srxn1*, and NRF2 targets from the combined analysis: *Areg, Tnc, Plaur* and *Angptl2*) and was subsequently used for *in vivo* experiments.

(**b**) Volcano plots of the differentially expressed (DE) genes in miR-NRF2 GBMs (n=3) compared with miR-ctl GBMs (n=3).

(**c**) GSEA ridge plots of the 10 most significant representative Hallmark gene lists.

(**d**) Low magnification of representative NRF2 IHC (brown) counterstained with hematoxylin (purple) on miR-ctl (n=4) and miR-NRF2 (n=4) GBM cryosections.

(**e**) Low magnification of representative SA-β-gal staining (blue) counter stained with hematoxylin (purple) on miR-ctl (n=4) and miR-NRF2 (n=4) GBM cryosections.

Scale bar, **d**, **e** 100 µm. **d**, **e**, Necrotic areas are outlined in red dashed lines. **b**, **c**: Analysis performed from bulk RNAseq of GBMs collected at the late time point. Raw data are provided as a Source Supplementary Data file.

Supplementary Figure 7

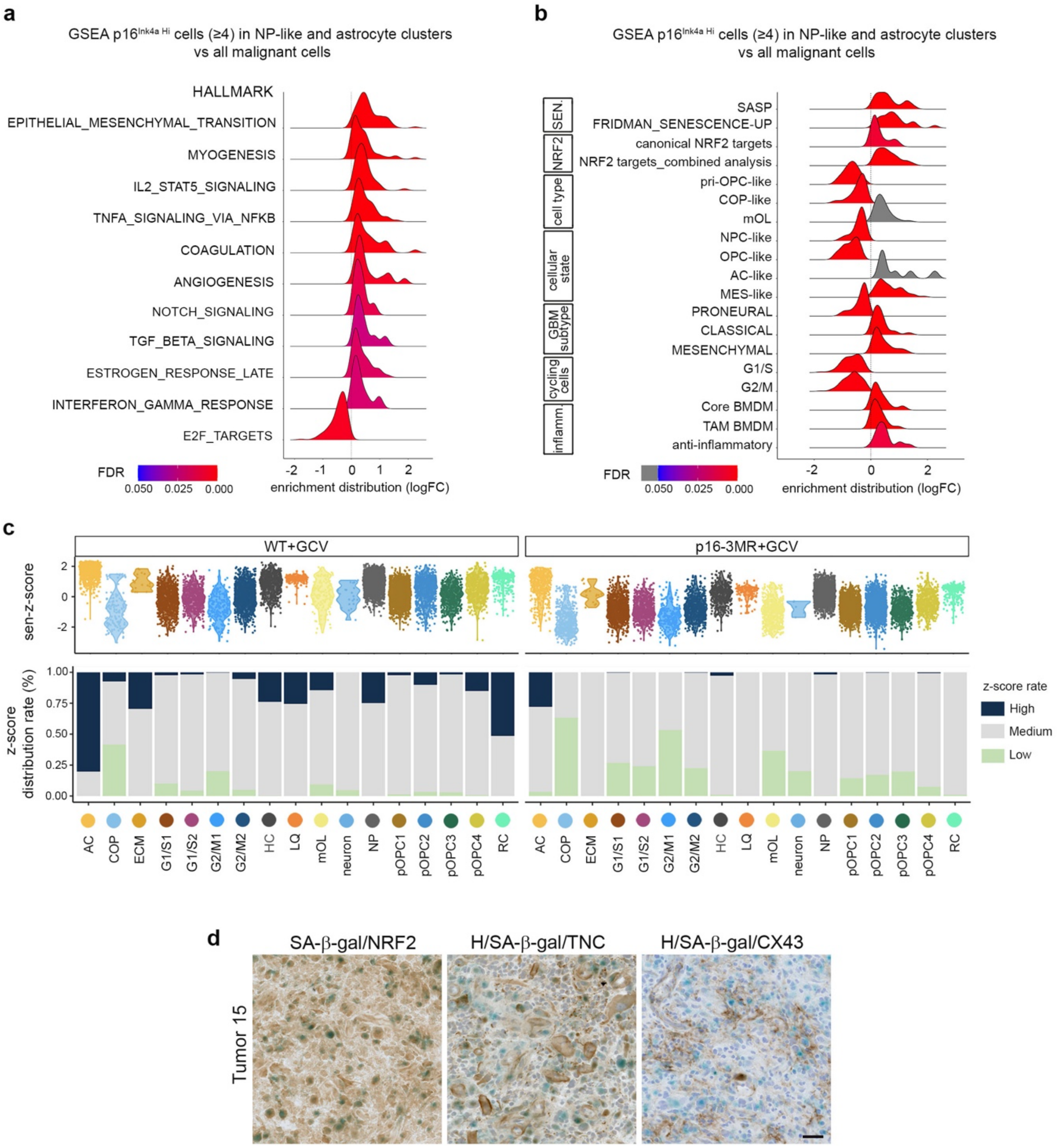

Supplementary Figure 7. Mouse senescent signature is conserved in patient GBM and its enrichment score is predictive of a worse survival

(**a**) GSEA ridge plot of significant Hallmark gene lists between the astrocyte and NP-like clusters compared with the remaining malignant cells in WT+GCV GBMs. The Hallmark gene lists represent the gene lists common to the GSEA analysis of the p16-3MR+GCV GBMs compared with controls (Supplementary Fig. 2e).

(**b**) GSEA ridge plot of gene lists used throughout the study (Supplementary Table 1), between the astrocyte and NP-like clusters compared with the remaining malignant cells in WT+GCV GBMs.

(**c**) Top: violin plots of the single sample GSEA (ssGSEA) senescent Z-score (sen-Z-score) in all malignant cells of WT+GCV and p16-3MR+GCV GBMs. Bottom: barplots of the percentage of the ssGSEA senescent Z-score distribution rate in all malignant cells of mouse GBMs. High and Low distribution rates correspond to the highest and lowest decile, respectively.

(**d**) Low magnification of representative SA-β-gal staining (blue) coupled with IHC (brown) and counterstained with hematoxylin (H, purple) on patient GBM cryosections. 3 patient GBMs were analyzed per antibody. Scale bar: 40 μm. Raw data are provided as a Source Supplementary Data file.
