# Supplementary material for: Cellular senescence in malignant cells promotes tumor progression in mouse and patient Glioblastoma
Source: Nat Commun. 2023 Jan 27;14:441. doi: 10.1038/s41467-023-36124-9 (PMC9883514; doi:10.1038/s41467-023-36124-9)
Supplement: Supplementary file 3 — Description of Additional Supplementary Files [file 41467_2023_36124_MOESM3_ESM.pdf]

Description of Additional Supplementary Files

Supplementary Data 1
Description:
- Gene lists used for GSEA
- MSigDB Gene lists used for GSEA
- FACs strategy. First, cells were selected from cellular debris based on the FSC area/SSC area; then singlets were selected based on the FSC area/ FSCHeight and FSC area / FSC weight. Finally, live cells were selected as negative for the Fixable Viability Dye Fluor. This last gating was determined with cells non-incubated with Fixable Viability Dye. (Related to Fig. 3b)

Supplementary Data 2
Description:
- Selected differentially expressed genes in CD45+ cells from p16-3MR-GBMs (pct.1) compared to WT+GCV GBMs (pct.2) (Early time point /10X scRNAseq/related to Fig. 4f) Statistical significance of the expression of genes in p16-3MR+GCV compared with WT+GCV clusters was determined by Wilcoxon-Mann-Whitney test (ns, not significant, *, p<0.05; **, p<0.01; ***, p<0.001).
- $p16^{Ink4a}$ fold change per CD45+ cluster in p16-3MR+GCV GBMs (pct.1) compared with WT+GCV GBMs (pct.2) (related to Fig.3h)

Supplementary Data 3
Description:
- Differentially downregulated genes in the p16-3MR+GCV vs the WT+GCV astrocyte cluster (Early time point/10X scRNAseq) avg-logFC ≤ -0.25; FDR ≤ 0.05 (related to Fig.5)
- Differentially upregulated genes in $p16^{Ink4a}$ positive vs $p16^{Ink4a}$ negative malignant cells (Late timepoint/ddSeq scRNAseq) avg-logFC ≥ 0.25; FDR ≤ 0.05 (related to Fig.5)
- Differentially down-regulated genes in the p16-3MR+GCV vs the WT+GCV GBMs (Late time point/Bulk RNAseq) avg-logFC ≤ -0.5; FDR ≤ 0.05 (related to Fig.5)
- NRF2 putative targets (related to Fig. 5)

Supplementary Data 4
Description:
- Univariate Cox analysis (related to Fig.7)
- Cox proportional hazards assumption (related to Fig.7)

Supplementary Data 5
Description:
- Antibodies for IHC/IF analysis
- Antibodies for WB analysis
- RT-qPCR primers
- miR oligonucleotides
- Suppliers for compounds
