# Supplementary material for: Cellular senescence in malignant cells promotes tumor progression in mouse and patient Glioblastoma
Source: Nat Commun. 2023 Jan 27;14:441. doi: 10.1038/s41467-023-36124-9 (PMC9883514; doi:10.1038/s41467-023-36124-9)
Supplement: Supplementary file 9 — Reporting Summary [file 41467_2023_36124_MOESM9_ESM.pdf]

nature portfolio

Corresponding author(s): Isabelle LE ROUX

Last updated by author(s): Dec 13, 2022

Reporting Summary

Nature Portfolio wishes to improve the reproducibility of the work that we publish. This form provides structure for consistency and transparency in reporting. For further information on Nature Portfolio policies, see our Editorial Policies and the Editorial Policy Checklist.

Statistics

For all statistical analyses, confirm that the following items are present in the figure legend, table legend, main text, or Methods section.

n/a	Confirmed	
☐	☒	The exact sample size (n) for each experimental group/condition, given as a discrete number and unit of measurement
☐	☒	A statement on whether measurements were taken from distinct samples or whether the same sample was measured repeatedly
☐	☒	The statistical test(s) used AND whether they are one- or two-sided<br>*Only common tests should be described solely by name; describe more complex techniques in the Methods section.*
☐	☒	A description of all covariates tested
☐	☒	A description of any assumptions or corrections, such as tests of normality and adjustment for multiple comparisons
☐	☒	A full description of the statistical parameters including central tendency (e.g. means) or other basic estimates (e.g. regression coefficient) AND variation (e.g. standard deviation) or associated estimates of uncertainty (e.g. confidence intervals)
☐	☒	For null hypothesis testing, the test statistic (e.g. F, t, r) with confidence intervals, effect sizes, degrees of freedom and P value noted<br>*Give P values as exact values whenever suitable.*
☒	☐	For Bayesian analysis, information on the choice of priors and Markov chain Monte Carlo settings
☒	☐	For hierarchical and complex designs, identification of the appropriate level for tests and full reporting of outcomes
☐	☒	Estimates of effect sizes (e.g. Cohen's d, Pearson's r), indicating how they were calculated

Our web collection on statistics for biologists contains articles on many of the points above.

Software and code

Policy information about availability of computer code

Data collection	Images were acquired using an Axio Scan.Z1 (Zeiss) and extracted using the ZEN 2.0 blue edition (Zeiss) software
Data analysis	Data analyses were performed using Fiji software version .2.1.0/1.53c; GraphPad Prism version 8.2.1; FastQC version 0.11.5; Star version 2.5.3a; rsem version 1.2.28; edgeR version 3.28.0; clusterProfiler version 3.14.3; Cell Ranger Single-cell Software suite version 3.0.2; Seurat bioconductor package version.3.2.3; R version 3.6; inferCNV package version 1.6.0; CellPhoneDB version 2.1.4; Seurat version 3.2.3; R package GSVA version 1.40.1 and version 1.32.0; survival R package version 2.44-1.1.<br>Computer codes used for the analysis of the senescence score are available in a supplementary Zip file.

For manuscripts utilizing custom algorithms or software that are central to the research but not yet described in published literature, software must be made available to editors and reviewers. We strongly encourage code deposition in a community repository (e.g. GitHub). See the Nature Portfolio guidelines for submitting code & software for further information.

Data

Policy information about availability of data

All manuscripts must include a data availability statement. This statement should provide the following information, where applicable:

- Accession codes, unique identifiers, or web links for publicly available datasets
- A description of any restrictions on data availability
- For clinical datasets or third party data, please ensure that the statement adheres to our policy

The mm10 reference genome was retrieved from https://hgdownload.cse.ucsc.edu/goldenpath/mm10/chromosomes/.
Gene Set Enrichment analysis gene sets came from MSigDB collections (https://software.broadinstitute.org/cancer/software/gsea/wiki/index.php/MSigDB_collections).
For the signature expression analysis on cells from patient GBMs, The Neftel et al., dataset was retrieved via the single cell portal (singlecell.broadinstitute.org), fastq files for the Bhaduri et al., dataset, were retrieved from SRA bioproject PRJNA579593, and the normalized expression matrix of Johnson and colleagues was retrieved via synapse (https://www.synapse.org/#!Synapse:syn22257780/wiki/604645).
Normalized intensities from TCGA microarray data were obtained from cBioPortal (cbioportal.org), filtering for GBM TCGA, Firehose Legacy dataset.
The raw data generated in this study are provided in a Source data file and a Source supplementary data file. The data generated in this study have been deposited in the Gene Expression Omnibus database under the accession code GSE168040. Further information and material requests should be addressed to Isabelle Le Roux (isabelle.leroux@icm-institute.org).

Human research participants

Policy information about studies involving human research participants and Sex and Gender in Research.

Reporting on sex and gender

SA-b-gal staining coupled to immunohistochemistry were performed on resected tissue coming from patients of both sex (n=11 female glioma; n=17 male glioma) and we did not find any association of the sex of the patient with a single senescent category as defined in the manuscript.

Population characteristics

Fresh patient GBM samples were selected from the Pitié-Salpêtrière tumor bank Onconeurotek. They were reviewed by our senior pathologist (Franck Bielle) to validate the histological features and confirm patients' diagnosis. Molecular characterizations were performed as previously described. The covariates, indicated in Supplementary Figure 1, include age, sex, tumor type according to the reference WHO classification, and additional molecular alterations including CDKN2A deletion status. Nine out of 28 patients were recruited at initial diagnosis and did not receive previous lines of treatment (radiotherapy or chemotherapy). Seven patients were recruited at recurrence and received first lines of treatment.

Recruitment

Consecutive patients, who had surgery for tumor resection in our institution, who had an appropriate tumor sample (sufficient quantity of fresh tumor tissue) and who gave their consent for research were recruited. Approximatively one half of glioblastomas widely infiltrate eloquent areas of the brain at the time of diagnosis and, thus, they cannot be resected and they have only a stereotactic needle biopsy, which correspond to an insufficient amount of tissue to perform this research project. So our study addressed only the subgroup of glioblastomas that can be resected. Our institution recruits only adult patients so we did not explore the biology of pediatric high grade gliomas which are rare distinct diseases.

Ethics oversight

Collection of tumor samples and clinical-pathological information were obtained upon patients' informed consent and ethical board approval, as stated by the Declaration of Helsinki. The ethical approval was obtained from the ethical committee « CPP – Ile de France VI – Groupe Hospitalier Pitié Salpêtrière ».

Note that full information on the approval of the study protocol must also be provided in the manuscript.

Field-specific reporting

Please select the one below that is the best fit for your research. If you are not sure, read the appropriate sections before making your selection.

☒ Life sciences ☐ Behavioural & social sciences ☐ Ecological, evolutionary & environmental sciences

For a reference copy of the document with all sections, see nature.com/documents/nr-reporting-summary-flat.pdf

Life sciences study design

All studies must disclose on these points even when the disclosure is negative.

Sample size

Our samples in this study are glioblastoma collected from a mouse model. We define the sample size by applying the 3R (Reduce, Replace, Refine) recommended for animal experimentation. Thus, we used the minimal sample size to ensure sufficient statistical power for the analysis.
For immunohistochemistry, SA-b-gal staining, RTqPCR and western blot analyses, no sample-size calculation was performed prior to experiment. However we subjected a minimum of n=4 tumors/condition for all of these experiments as statistical significance was determined by a two-sided unpaired Wilcoxon-Mann-Whitney test. Indeed, the minimum sample size to obtain a statistical significance with this test is 4 samples per condition.

For the survival analyses, at the beginning of the study, we could not assume any effect size of senolytics on GBM. Thus, we estimated the minimum sample size by the method called "resource equation" (Charan et al. 2013). According to this method, we needed between 6 to 11 animals per treatment group to ensure an adequate statistical power. Retrospectively, we observed a 15% increase of survival in our treated group. We thus applied the parameters a=0.05, beta=0.2 on the UCSF sample size calculator (Sample size – Survival analysis | Sample Size Calculators (sample-size.net)) which estimated the minimal number to 9 mice / group.

Data exclusions

Survival analysis. We did exclude some animals from the survival analysis when they did not show any tumor growth (bioluminescence signal) after 100 days post lentivirus (coding for H-RasV12-shp53) injection. Mice were injected with lentivirus by batch. One batch always included control and experimental mice injected the same day. When control mice survival extended more than 57 DPI in the paradigm without bioluminescence monitoring (Fig. 2a), the entire batch was removed from the analysis to exclude technical bias linked to intracranial injection.

Bulk RNAseq analysis. For the RNAseq analysis at late timepoint 2/9 p16-3MR+GCV samples were removed from the analysis of p16-3MR+GCV vs p16-3MR+vhc GBMs based on the PCA analysis.

Replication

Whenever it was feasible, we replicated the different analysis. Each sample was triplicated for qPCR experiments. Western blot experiment was replicated twice. Immunohistochemistry was performed on a minimum of 4 samples / condition. Survival analyses with the p16-3MR transgene was replicated twice using one distinct control each time (WT+GCV or p16-3MR+vhc). Survival analyses with the ABT263 vs vehicule and miRcontrol vs miRNRF2 were performed only once. As these analyses displayed a statistical significance determined by Mantel-Cox log-rank test, we did not replicate these studies. RNAseq experiments were performed on a minimum of 3 samples / condition.

Randomization

All samples of the same genotype were randomly allocated into experimental groups

Blinding

The investigators were blinded to group allocation (control cohort vs senolytic treated cohort; control cohort vs mirNRF2 cohort) during data analysis (western blot analysis; immunohistochemistry and SA-b-gal staining) except for survival curves as mice were all identified by a number to ensure proper treatment administration.

Reporting for specific materials, systems and methods

We require information from authors about some types of materials, experimental systems and methods used in many studies. Here, indicate whether each material, system or method listed is relevant to your study. If you are not sure if a list item applies to your research, read the appropriate section before selecting a response.

Materials & experimental systems

n/a	Involved in the study
☐	☒ Antibodies
☐	☒ Eukaryotic cell lines
☒	☐ Palaeontology and archaeology
☐	☒ Animals and other organisms
☒	☐ Clinical data
☒	☐ Dual use research of concern

Methods

n/a	Involved in the study
☒	☐ ChIP-seq
☐	☒ Flow cytometry
☒	☐ MRI-based neuroimaging

Antibodies

Antibodies used

IMMUNOHISTOCHEMISTRY
- anti-Ki67 Rabbit monoclonal (SP6) dilution 1/100 Vector Laboratories Cat# VP-RM04, RRID:AB_2336545
- anti-LAMIN-B1 Mouse IgG1 (B-10) dilution 1/500 Santa Cruz Biotechnology Cat# sc-374015, RRID:AB_10947408
- anti-GFAP Mouse IgG1 (5G-A-5) dilution 1/2000 Sigma-Aldrich Cat# G3893, RRID:AB_477010
- anti-OLIG2 Rabbit polyclonal dilution1/1000 Millipore Cat# AB9610, RRID:AB_570666
- anti-IBA1 Rabbit polyclonal dilution 1/800 Wako Cat# 019-19741, RRID:AB_839504
- anti-GFP Chicken polyclonal dilution 1/2000 Aves Labs Cat# GFP-1020, RRID:AB_10000240
- anti-p19 Rat monoclonal (PIL346C) dilution 1/2 CNIO #340C/B3
- anti-p16 Rabbit monoclonal [EPR1473] dilution 1/200 abcam Cat# ab108349, RRID:AB_10858268
- anti-p53 Mouse monoclonal (DO-7) dilution 1/500 Agilent DAKO Cat# GA616 RRID:AB_2889978
- anti-CD31 Rat monoclonal dilution (MEC 7.46) 1/200 BD Bioscience Cat# GTX27388, RRID:AB_369216
- anti-NRF2 Rabbit polyclonal dilution 1/500) (mouse) or 1/100 (human) Thermo Fisher Scientific Cat# PA5-27882, RRID:AB_2545358
- anti-TNC Mouse IgG1 (4C8MS) dilution 1/250 Novus Cat# NB110-68136, RRID:AB_1110904
- anti-CX43 Rabbit polyclonal dilution 1/50 Cell Signaling Technology Cat# 3512, RRID:AB_2294590
- anti-uPAR Goat polyclonal dilution 1/100 R and D Systems Cat# AF534, RRID:AB_2165351

- Goat anti-mouse (1/500) Vector Laboratories Cat# BP-9200, RRID:AB_2827937
- Goat anti-mouse IgG1 (1/500) Thermo Fisher Scientific Cat# A10519, RRID:AB_2534028
- Rabbit anti-rat (1/500) Vector Laboratories Cat# BA-4001, RRID:AB_10015300
- Goat anti-rabbit (1/500) Vector LaborDO-7)atories Cat# PI-1000, RRID:AB_2336198
- Goat anti-chicken (1/500) Vector Laboratories Cat# BA-9010, RRID:AB_2336114

WESTERN-BLOT
- anti-TNC Mouse IgG1 (4C8MS) dilution 1/200 Novus Cat# NB110-68136, RRID:AB_1110904
- anti-β-TUBULIN Mouse IgG1 (TUB 2.1) dilution 1/10 000 Sigma-Aldrich Cat# T4026, RRID:AB_477577

- anti-Mouse IgG DL800 Mouse dilution 1/10 000 Cell Signaling Cat# 5257, RRID:AB_10693543
- anti-Mouse IgG DL680 Mouse dilution 1/10 000 Cell Signaling Cat# 5470 RRID:AB_10696895

Validation	All antibodies were validated by testing the secondary antibodies alone. For immunohistochemistry, the correct cellular localisation of the signal (membrane bound, nuclear, cytoplasmic) or for western blot, the correct size of the signal further validated the antibodies. Validation statements for each of the antibodies used in the study are on the manufacturer's website.

Eukaryotic cell lines

Policy information about cell lines and Sex and Gender in Research

Cell line source(s)	Glioma 261 cell line (GL261) was retrieved from DSMZ (#ACC 802). Sex was not reported for this cell line derived from murine (C57BL/6 mouse) glioblastoma.
Authentication	GL261 cell line was not authenticated.
Mycoplasma contamination	GL261 cell line was tested negative for mycoplasma contamination.
Commonly misidentified lines (See ICLAC register)	No commonly misidentified cell lines were used in the study.

Animals and other research organisms

Policy information about studies involving animals; ARRIVE guidelines recommended for reporting animal research, and Sex and Gender in Research

Laboratory animals	To generate our GBM mouse model, we crossed GlastcreERT2/+ mice with the Ptenfl/fl mice. GlastcreERT2/+; Ptenfl/fl males were bred with either Ptenfl/fl or Ptenfl/fl; p16-3MR/+ females to generate GlastcreERT2/+; Ptenfl/fl and GlastcreERT2/+; Ptenfl/fl; p16-3MR/+ mice. All animals used in the study were 6-8 week-old female mice at inclusion except for the mice used for scRNAseq at the early timepoint that were 14-week-old mice.
Wild animals	The study did not involve wild animals
Reporting on sex	Our animal study was performed only on female mice. In the present study, the analysis on the senescence Z-score, based on our scRNAseq analysis on mouse GBM cells and applied to patient GBM TCGA data showed that regardless of the sex of the patient, the enrichment of the senescence Z-score predicted a worse survival to patients with GBM. Thus, this retrospective analysis validated our initial choice to work only on one sex.
Field-collected samples	No field collected samples were used in the study.
Ethics oversight	All animal care and treatment protocols complied with European legislation (no. 2010/63/UE) and national (French Ministry of Agriculture) guidelines for the use and ethical treatment of laboratory animals. All experiments on animals were approved by the ethical committee in animal experimentation Charles Darwin n°5, Paris (approval APAFIS 9131).

Note that full information on the approval of the study protocol must also be provided in the manuscript.

Flow Cytometry

Plots

Confirm that:

☒ The axis labels state the marker and fluorochrome used (e.g. CD4-FITC).

☒ The axis scales are clearly visible. Include numbers along axes only for bottom left plot of group (a 'group' is an analysis of identical markers).

☒ All plots are contour plots with outliers or pseudocolor plots.

☒ A numerical value for number of cells or percentage (with statistics) is provided.

Methodology

Sample preparation	After brain harvest, GFP+ tumors were dissected under a Leica MZFL II stereomicroscope. Tumor pieces were chopped and incubated 5 min at 37°C in a HBSS-papain based lysis buffer (Worthington PAP) containing DNAse (0.01%, Worthington #LS002139) and L-Cystein (124 µg/mL, Sigma #C78805). Papain digestion was inhibited by ovomucoid (7 mg/mL, Worthington #LS003085). Tissue was further dissociated mechanically and centrifuged 300 g, 10 min at 4°C. Cells were resuspended in cold HBSS, a debris removal step was performed (Miltenyi #130-109-398) and blood cells were removed using a blood lysis buffer (Roche 11814 389001). After centrifugation, cells were resuspended in cold HBSS and incubated with the eBiosciences Fixable Viability Dye Fluor 450 or 660 (Invitrogen 65-0863), to label dead cells, and washed. Cells were then sorted using the MoFlo Astrios cell sorter (Beckman Coulter) or the S3e cell sorter (Biorad). Live cells were collected in HBSS 0.1% BSA precoated tubes.
Instrument	S3e cell sorter (Biorad) or MoFlo Astrios cell sorter (Beckman Coulter)

Software	The flow cytometry data were analyzed during the cell sorting using the sofware from Biorad (Prosort) or Beckman Coulter (Summit).
Cell population abundance	Live cells represented > 90% of cells previously identified as singlets.
Gating strategy	First, cells were selected from cellular debris based on the FSC area/SSC area; then singlets were selected based on the FSC area/ FSCHeight and FSC area / FSC weight. Finally, live cells were selected as negative for the Fixable Viability Dye Fluor. This last gating was determined with cells non-incubated with Fixable Viability Dye.

☒ Tick this box to confirm that a figure exemplifying the gating strategy is provided in the Supplementary Information.
